# Chemolithotrophy in the continental deep subsurface: Sanford Underground Research Facility (SURF), USA

**DOI:** 10.3389/fmicb.2014.00610

**Published:** 2014-11-12

**Authors:** Magdalena R. Osburn, Douglas E. LaRowe, Lily M. Momper, Jan P. Amend

**Affiliations:** ^1^Department of Earth Sciences, University of Southern CaliforniaLos Angeles, CA, USA; ^2^Department of Earth and Planetary Sciences, Northwestern UniversityEvanston, IL, USA; ^3^Department of Biological Sciences, University of Southern CaliforniaLos Angeles, CA, USA

**Keywords:** energetics, deep subsurface biosphere, SURF, chemolithotrophy, biogeochemistry

## Abstract

The deep subsurface is an enormous repository of microbial life. However, the metabolic capabilities of these microorganisms and the degree to which they are dependent on surface processes are largely unknown. Due to the logistical difficulty of sampling and inherent heterogeneity, the microbial populations of the terrestrial subsurface are poorly characterized. In an effort to better understand the biogeochemistry of deep terrestrial habitats, we evaluate the energetic yield of chemolithotrophic metabolisms and microbial diversity in the Sanford Underground Research Facility (SURF) in the former Homestake Gold Mine, SD, USA. Geochemical data, energetic modeling, and DNA sequencing were combined with principle component analysis to describe this deep (down to 8100 ft below surface), terrestrial environment. SURF provides access into an iron-rich Paleoproterozoic metasedimentary deposit that contains deeply circulating groundwater. Geochemical analyses of subsurface fluids reveal enormous geochemical diversity ranging widely in salinity, oxidation state (ORP 330 to −328 mV), and concentrations of redox sensitive species (e.g., Fe^2+^ from near 0 to 6.2 mg/L and Σ S^2-^ from 7 to 2778μg/L). As a direct result of this compositional buffet, Gibbs energy calculations reveal an abundance of energy for microorganisms from the oxidation of sulfur, iron, nitrogen, methane, and manganese. Pyrotag DNA sequencing reveals diverse communities of chemolithoautotrophs, thermophiles, aerobic and anaerobic heterotrophs, and numerous uncultivated clades. Extrapolated across the mine footprint, these data suggest a complex spatial mosaic of subsurface primary productivity that is in good agreement with predicted energy yields. Notably, we report Gibbs energy normalized both per mole of reaction and per kg fluid (energy density) and find the later to be more consistent with observed physiologies and environmental conditions. Further application of this approach will significantly expand our understanding of the deep terrestrial biosphere.

## Introduction

A majority of the microbial life on Earth may reside in subsurface environments. The total integrated mass of the marine subsurface biosphere has recently been estimated at 1.5–22 petagrams carbon (Pg C) (Hinrichs and Inagaki, 2012; Kallmeyer et al., 2012), a significant downward revision from older estimates (Whitman et al., 1998). A recent review by McMahon and Parnell (2013) has similarly evaluated the size of the terrestrial deep subsurface biosphere (DSB), incorporating new study locations, cell density estimates, porosity data, and carbon content of cells, converging on an estimate of 14–135 Pg C (McMahon and Parnell, 2013). Notably, this estimate is larger than or on par with those from marine sediments (1.5–22 Pg C, Kallmeyer et al., 2012), terrestrial soil (26 Pg C, Whitman et al., 1998), or aquatic environments (2.2 Pg C, Whitman et al., 1998); however, the range is very large. Recent efforts focused on the study of terrestrial subsurface sites are providing data that can be used to better determine the total mass of the terrestrial subsurface biosphere and its connection to the surface world. For example, billion year old water and associated ecosystems in the Canadian Shield have been described (Holland et al., 2013), a monophyletic community in deep South African gold mines has been reported (Chivian et al., 2008), and life in seemingly impossible ultrabasic conditions now seems likely (Brazelton et al., 2012).

In the current study, microbiological and geochemical data are presented from a new portal into the deep terrestrial biosphere, the Sanford Underground Research Laboratory (SURF) in the former Homestake Gold Mine, South Dakota USA. The Homestake Gold Mine, active from 1876 to 2001, produced 1101 tons of gold from tunnels as deep as 8100 ft (Caddey, 1991). After mining activity ceased, the site was transformed into a state-run science facility primarily focused on particle physics. Mining tunnels intersect three Paleoproterozoic metasedimentary units, the Poorman, Homestake, and Ellison formations, with exploratory boreholes extending well beyond the primary mining footprint (Caddey, 1991). Hydrological modeling indicates relatively shallow meteoric input in upper mining levels and much older (>10,000 yrs) fluids reaching the deeper levels, especially on the northern ledges (Murdoch et al., 2011). Previous microbiological studies at SURF have focused on the mine tunnel environment, primarily for the identification of industrially-relevant cellulose degrading bacteria (Rastogi et al., 2009, 2010, 2013). For the purpose of this study, the mine serves as access to the *in situ* subsurface biosphere, i.e., habitats that have been least affected by mining activity.

The hydrological and geological variability present in the continental subsurface can be much greater than in marine environments. For instance, the continental crust is composed of a combination of felsic and mafic, intrusive and extrusive, igneous rocks and sedimentary deposits of numerous compositions and metamorphic grades. Consequently, a multifaceted approach is necessary to characterize the environmental complexities of deep terrestrial ecosystems, including understanding the feeding aquifer and the host lithologies encountered by the corresponding subsurface fluids. Such information is often available in mines because it is critical to the structural safety and economics of mining. The chemistry of subsurface fluids reflects interaction with different rock lithologies, and thus differs depending on sample location and depth. The variable salinities, pHs, temperatures, and oxidation states will, in turn, support different microbial communities that can be probed with biomolecular techniques, including pyrosequencing. Although these techniques have limited utility in connecting sequence identity to function, they can be used to rapidly assess the microbial diversity of an environment and to infer putative catabolic strategies. Thermodynamic modeling can then be used in concert with geochemical and genomic data to ascertain likely ongoing chemolithotrophic strategies. Here, we combined these techniques and present a characterization of the geochemical habitat, microbiome, and energetic framework for chemolithotrophic processes of the subsurface biosphere at SURF.

## Materials and methods

### Field and laboratory measurements

The samples for this study were acquired over the course of three expeditions to SURF in Lead, South Dakota (USA) in Sept. 2013, Oct. 2013, and Feb. 2014 (Yates Shaft, latitude 44.352157**°**, longitude -103.750503**°**). Detailed geochemical data were acquired only on the last two trips. Oxidation-reduction potential (ORP), conductivity, pH, temperature, and total dissolved solids (TDS) were measured *in situ* with an Ultrameter II 6PFC^E^ (Myron L Company). Redox sensitive species (DO, ∑S^2−^, Fe^++^, Mn^2+^, NO^−^_3_, NO^−^_2_, NH^+^_4_, SiO_2_, ∑PO^−3^_4_) were measured using Hach DR/2400 portable field spectrophotometers and associated reaction kits (Hack Company, Loveland, CO).

Samples for major anions, cations, and dissolved gasses were preserved for laboratory analysis. Major anions were measured using a Metrohm 850 Professional Ion Chromatograph equipped with are Metrosep A Supp 5, 250 × 4 mm column and conductivity detector. The mobile phase was 3.2 mM Na_2_CO_3_, 1.0 mM NaHCO_3_, and 2.5% acetonitrile following at 0.7 ml/min. Cations were measured using an Agilent 4100 Microwave Plasma Atomic Emission Spectrometer. Samples were diluted 1:10 or 1:100 in 5% HNO_3_, and concentrations were calculated relative to synthetic standards prepared in the same manner. Dissolved gas samples were collected by either the bubble stripping method (Alter and Steiof, 2005) where in-line filtering was possible (sites B, D, 6, 9), or via bulk fluid collection into evacuated serum vials (2, 3A 5, 8). The bubble stripping method has significantly higher sensitivity and is preferable when possible. Headspace concentrations of He, H_2_, O_2_, N_2_, CO_2_, CH_4_, CO, ethane, and propane were measured with a Shimadzu GC-2014ATF headspace GC equipped with Haysep 80/100 (5 m) and MS-5A 60/80 (2.5 m) molecular sieve columns and TCD and FID detectors. Dissolved gas concentrations were calculated from headspace gas concentrations based on solubility constants of each gas at the analytical temperature and pressure and comparison to standard gas mixtures.

### Thermodynamic modeling

Geochemical data from each site were used to calculate the Gibbs energy yields of 140 potential catabolic reactions. These reactions coupled the electron acceptors O_2_, NO^−^_3_, MnO_2_, Fe_3_O_4_, SO^−2^_4_, and S^0^ with the electron donors Fe^++^, CH_4_, H_2_, S^0^, NH_4_^+^, HS^−^, CO, and Mn^++^ (Table 1). Values of Gibbs energy yields were calculated using

(1) $$\Delta G_{r}=-RT\ln\frac{K_{r}}{Q_{r}}$$

where Δ*G_r_* represents the Gibbs energy of reaction *r* under *in situ* conditions, *K_r_* and *Q_r_* stand for the equilibrium constant and activity product of the *r*th reaction, *R* refers to the gas constant, and *T* denotes temperature in kelvin. Values of *K_r_* were calculated using the revised-HKF equations of state (Helgeson et al., 1981; Tanger and Helgeson, 1988; Shock et al., 1992), the SUPCRT92 software package (Johnson et al., 1992), and thermodynamic data taken from Bricker (1965), Hem et al. (1982), Shock and Helgeson (1988, 1990); Shock et al. (1989), Sverjensky et al. (1997), Schulte et al. (2001). Values of *Q_r_* for each reaction were calculated from

(2) $$Q_{r}=\prod a_{i}^{v_{i,r}}$$

where *a_i_* designates the activity of the *i*th species and *v_i,r_* indicates the stoichiometric coefficient of the *i*th species in the *r*th reaction. Activities were determined using geochemical data from each field site and the program SPEC8 (Geochemist's Workbench 8, Aqueous Solutions LLC). Δ*G_r_* is presented in units of kilojoules per mole of electron transferred, kJ (mol e^−^)^−1^. In order to scale energy availability to the limiting reactant, the Gibbs energy calculations are also presented in terms of energy densities, *E_r_*, which are calculated by

(3) $$E_{r}=\left|\frac{△G_{r}}{v_{i}}\right|\left[i\right]$$

where [*i*] refers to the concentration of the limiting electron donor or acceptor (LaRowe and Amend, 2014). The metric represented by Equation (3) has been shown to correlate with biomass abundance better than the typical reporting of Δ*G_r_* in molar units (LaRowe and Amend, 2014).

**Table 1:** **Reactions considered in this study**.

**No**.	**Reaction**	**e-/rxn**
**O_2_ AS ELECTRON ACCEPTOR**		
1	O_2_^*^ + 2H_2_ ↔ 2H_2_O	4
2	O_2_ + 4Fe^++^ + 6H_2_O ↔ 4FeOOH_fer_^**^ + 8H^+^	4
3	O_2_ + 4Fe^++^ + 6H_2_O ↔ 4FeOOH_goe_ + 8H^+^	4
4	O_2_ + 6Fe^++^ + 6H_2_O ↔ 2Fe_3_O_4_ + 12H^+^	4
5	O_2_ + 4Mn^++^ + 6H_2_O ↔ 4MnOOH_feit_ + 8H^+^	4
6	O_2_ + 4Mn^++^ + 6H_2_O ↔ 4MnOOH_man_ + 8H^+^	4
7	O_2_ + 2Mn^++^ + 2H_2_O ↔ 2MnO_2_ + 4H^+^	4
8	3O_2_ + 4NH_4_^+^ ↔ 6H_2_O + 2N_2_ + 4H^+^	12
9	2O_2_ + NH_4_^+^ ↔ NO^−^_3_ + 2H^+^ + H_2_O	8
10	O_2_ + 2H^+^ + 2HS^−^ ↔ 2S^0^ + 2H_2_O	4
11	2O_2_ + HS^−^ ↔ SO_4_^−2^ + H^+^	8
12	3O_2_ + 2S^0^ + 2H_2_O ↔ 2SO_4_^−2^ + 4H^+^	12
13	3O_2_ + 2CH_4_ ↔ 2CO + 4H_2_O	12
14	2O_2_ + CH_4_ ↔ HCO^−^_3_ + H^+^ + H_2_O	8
15	O_2_ + 2CO + 2H_2_O ↔ 2HCO^−^_3_ + 2H^+^	4
**NO^−3^ AS ELECTRON ACCEPTOR**		
16	2NO^−^_3_ + 2H^+^ + 5H_2_ ↔ N_2_ + 6H_2_O	10
17	2NO^−^_3_ + 10Fe^++^ + 14H_2_O ↔ N_2_ + 10FeOOH_fer_ + 18H^+^	10
18	2NO^−^_3_ + 10Fe^++^ + 14H_2_O ↔ N_2_ + 10FeOOH_goe_ + 18H^+^	10
19	2NO^−^_3_ + 15Fe^++^ + 14H_2_O ↔ 5Fe_3_O_4_ + 28H^+^ + N_2_	10
20	2NO^−^_3_ + 10Mn^++^ + 14H_2_O ↔ 10MnOOH_feit_ + 18H^+^ + N_2_	10
21	2NO^−^_3_ + 10Mn^++^ + 14H_2_O ↔ 10MnOOH_man_ + 18H^+^ + N_2_	10
21	2NO^−^_3_ + 5Mn^++^ + 4H_2_O ↔ N_2_ + 5MnO_2_ + 8H^+^	10
23	2NO^−^_3_ + 7H^+^ + 5HS^−^ ↔ 5S^0^ + N_2_ + 6H_2_O	10
24	8NO^−^_3_ + 3H^+^ + 5HS^−^ ↔ 5SO_4_^−2^ + 4N_2_ + 4H_2_O	40
25	6NO^−^_3_ + 5S^0^ + 2H_2_O ↔ 5SO_4_^−2^ + 4H^+^ + 3N_2_	30
26	6NO^−^_3_ + 6H^+^ + 5CH_4_ ↔ 5CO + 3N_2_ + 13H_2_O	30
27	8NO^−^_3_ + 3H^+^ + 5CH_4_ ↔ 5HCO^−^_3_ + 4N_2_ + 9H_2_O	40
28	2NO^−^_3_ + 5CO + 4H_2_O ↔ 5HCO^−^_3_ + 3H^+^ +N_2_	10
29	NO^−^_3_ + 2H^+^ + 4H_2_ ↔ NH_4_^+^ + 3H_2_O	8
30	NO^−^_3_ + 8Fe^++^ + 13H_2_O ↔ NH_4_^+^ + 8FeOOH_fer_ + 14H^+^	8
31	NO^−^_3_ + 8Fe^++^ + 13H_2_O ↔ NH_4_^+^ + 8FeOOH_goe_ + 14H^+^	8
32	NO^−^_3_ + 12Fe^++^ + 13H_2_O ↔ 4Fe_3_O_4_ + 22H^+^ + NH_4_^+^	8
33	NO^−^_3_ + 8Mn^++^ + 13H_2_O ↔ 8MnOOH_feit_ + 14H^+^ + NH4^+^	8
34	NO^−^_3_ + 8Mn^++^ + 13H_2_O ↔ 8MnOOH_man_ + 14H^+^ + NH_4_^+^	8
35	NO^−^_3_ + 4Mn^++^ + 5H_2_O ↔ 4MnO_2_ + 6H^+^ + NH_4_^+^	8
36	NO^−^_3_ + 6H^+^ + 4HS^−^ ↔ 4S^0^ + NH_4_^+^ + 3H_2_O	8
37	NO^−^_3_ + H^+^ + HS^−^ + H_2_O ↔ SO_4_^−2^ + NH_4_^+^	8
38	3NO^−^_3_ + 4S^0^ + 7H_2_O ↔ 4SO_4_^−2^ + 2H^+^ + 3NH_4_^+^	24
39	3NO^−^_3_ + 6H^+^ + 4CH_4_ ↔ 4CO + 3NH_4_^+^ + 5H_2_O	24
40	NO^−^_3_ + H^+^ + CH_4_ ↔ HCO^−^_3_ + NH_4_^+^	8
41	NO^−^_3_ + 4CO + 5H_2_O = 4HCO^−^_3_ + 2H^+^ + NH_4_^+^	8
**SO^−2^_4_AS ELECTRON ACCEPTOR**		
42	SO_4_^−2^ + H^+^ + 4H_2_ ↔ HS^−^ + 4H_2_O	8
43	SO_4_^−2^ + 8Fe^++^ + 12H_2_O ↔ 8FeOOH_fer_ + 15H^+^ + HS^−^	8
44	SO_4_^−2^ + 8Fe^++^ + 12H_2_O ↔ 8FeOOH_goe_ + 15H^+^ + HS^−^	8
45	SO_4_^−2^ + 12Fe^++^ + 12H_2_O ↔ 4Fe_3_O_4_ + 23H^+^ + HS^−^	8
46	SO_4_^−2^ + 8Mn^++^ + 12H_2_O ↔ 8MnOOH_feit_ + 15H^+^ + HS^−^	8
47	SO_4_^−2^ + 8Mn^++^ + 12H_2_O ↔ 8MnOOH_man_ + 15H^+^ + HS^−^	8
48	SO_4_^−2^ + 4Mn^++^ + 4H_2_O ↔ 4MnO_2_ + 7H^+^ + HS^−^	8
49	3SO_4_^−2^ + 8NH_4_^+^ ↔ 4N_2_ + 5H^+^ + 3HS^−^ + 12H_2_O	24
50	SO_4_^−2^ + NH_4_^+^ ↔ NO^−^_3_ + H^+^ + HS^−^ + H_2_O	8
51	3SO_4_^−2^ + 3H^+^ + 4CH_4_ ↔ 4CO + 3HS^−^ + 8H_2_O	24
52	SO_4_^−2^ + CH_4_ ↔ HCO^−^_3_ + HS^−^ + H_2_O	8
53	SO_4_^−2^ + 4CO + 4H_2_O ↔ 4HCO^−^_3_ + 3H^+^ + HS^−^	8
54	SO_4_^−2^ + 2H^+^ + 3H_2_ ↔ S^0^ + 4H_2_O	6
55	SO_4_^−2^ + 6Fe^++^ + 8H_2_O ↔ 6FeOOH_fer_ + 10H^+^ + S^0^	6
56	SO_4_^−2^ + 6Fe^++^ + 8H_2_O ↔ 6FeOOH_goe_ + 10H^+^ + S^0^	6
57	SO_4_^−2^ + 9Fe^++^ + 8H_2_O ↔ 3Fe_3_O_4_ + 16H^+^ + S^0^	6
58	SO_4_^−2^ + 6Mn^++^ + 8H_2_O ↔ 6MnOOH_feit_ + 10H^+^ + S^0^	6
59	SO_4_^−2^ + 6Mn^++^ + 8H_2_O ↔ 6MnOOH_man_ + 10H^+^ + S^0^	6
60	SO_4_^−2^ + 3Mn^++^ + 2H_2_O ↔ 3MnO_2_ + 4H^+^ + S^0^	6
61	SO_4_^−2^ + 2NH_4_^+^ ↔ N_2_ + S^0^ + 4H_2_O	6
62	4SO_4_^−2^ + 2H^+^ + 3NH_4_^+^ ↔ 3NO^−^_3_ + 4S^0^ + 7H_2_O	24
63	SO_4_^−2^ + 5H^+^ + 3HS^−^ ↔ 4S^0^ + 4H_2_O	6
64	SO_4_^−2^ + 2H^+^ + CH_4_ ↔ CO + S^0^ + 3H_2_O	6
65	4SO_4_^−2^ + 5H^+^ + 3CH_4_ ↔ 3HCO^−^_3_ + 4S^0^ + 7H_2_O	24
66	SO_4_^−2^ + 3CO + 2H_2_O ↔ 3HCO^−^_3_ + H^+^ + S^0^	6
**ELEMENTAL SULFUR AS ELECTRON ACCEPTOR**		
67	S^0^ + H_2_ ↔ H^+^ + HS^−^	2
68	S^0^ + 2Fe^++^ + 4H_2_O ↔ 2FeOOH_fer_ + 5H^+^ + HS^−^	2
69	S^0^ + 2Fe^++^ + 4H_2_O ↔ 2FeOOH_goe_ + 5H^+^ + HS^−^	2
70	S^0^ + 3Fe^++^ + 4H_2_O ↔ Fe_3_O_4_ + 7H^+^ + HS^−^	2
71	S^0^ + 2Mn^++^ + 4H_2_O ↔ 2MnOOH_feit_ + 5H^+^ + HS^−^	2
72	S^0^ + 2Mn^++^ + 4H_2_O ↔ 2MnOOH_man_ + 5H^+^ + HS^−^	2
73	S^0^ + Mn^++^ + 2H_2_O ↔ MnO_2_ + 3H^+^ + HS^−^	2
74	3S^0^ + 2NH_4_^+^ ↔ N_2_ + 5H^+^ + 3HS^−^	6
75	4S^0^ + NH_4_^+^ + 3H_2_O ↔ NO^−^_3_ + 6H^+^ + 4HS^−^	8
76	4S^0^ + 4H_2_O ↔ SO_4_^−2^ + 3HS^−^ + 5H^+^	6
77	3S^0^ + CH_4_ + H_2_O ↔ CO + 3H^+^ + 3HS^−^	6
78	4S^0^ + CH_4_ + 3H_2_O ↔ HCO^−^_3_ + 5H^+^ + 4HS^−^	8
79	S^0^ + CO + 2H_2_O ↔ HCO^−^_3_ + 2H^+^ + HS^−^	2
**CARBON MONOXIDE AS ELECTRON ACCEPTOR**		
80	CO + 3H_2_ ↔ CH_4_ + H_2_O	6
81	CO + 6Fe^++^ + 11H_2_O ↔ 6FeOOH_fer_ + 12H^+^ + CH_4_	6
82	CO + 6Fe^++^ + 11H_2_O ↔ 6FeOOH_goe_ + 12H^+^ + CH_4_	6
83	CO + 9Fe^++^ + 11H_2_O ↔ 3Fe_3_O_4_ + 18H^+^ + CH_4_	6
84	CO + 6Mn^++^ + 11H_2_O ↔ 6MnOOH_feit_ + 12H^+^ + CH_4_	6
85	CO + 6Mn^++^ + 11H_2_O ↔ 6MnOOH_man_ + 12H^+^ + CH_4_	6
86	CO + 3Mn^++^ + 5H_2_O ↔ 3MnO_2_ + 6H^+^ + CH_4_	6
87	CO + 2NH_4_^+^ ↔ N_2_ + 2H^+^ + CH_4_ + H_2_O	6
88	4CO + 3NH_4_^+^ + 5H_2_O ↔ 3NO^−^_3_ + 6H^+^ + 4CH_4_	24
89	CO + 3H^+^ + 3HS^−^ ↔ 3S^0^ + CH_4_ + H_2_O	6
90	4CO + 3HS^−^ + 8H_2_O ↔ 3SO_4_^−2^ + 3H^+^ + 4CH_4_	24
91	CO + S^0^ + 3H_2_O ↔ SO_4_^−2^ + 2H^+^ + CH_4_	6
**BICARBONATE AS ELECTRON ACCEPTOR**		
92	HCO^−^_3_ + H^+^ + 4H_2_ ↔ CH_4_ + 3H_2_O	8
93	HCO^−^_3_ + 8Fe^++^ + 13H_2_O ↔ 8FeOOH_fer_ + 15H^+^ + CH_4_	8
94	HCO^−^_3_ + 8Fe^++^ + 13H_2_O ↔ 8FeOOH_goe_ + 15H^+^ + CH_4_	8
95	HCO^−^_3_ + 12Fe^++^ + 13H_2_O ↔ 4Fe_3_O_4_ + 23H^+^ + CH_4_	8
96	HCO^−^_3_ + 8Mn^++^ + 13H_2_O ↔ 8MnOOH_feit_ + 15H^+^ + CH_4_	8
97	HCO^−^_3_ + 8Mn^++^ + 13H_2_O ↔ 8MnOOH_man_ + 15H^+^ + CH_4_	8
98	HCO^−^_3_ + 4Mn^++^ + 5H_2_O ↔ 4MnO_2_ + 7H^+^ + CH_4_	8
99	3HCO^−^_3_ + 8NH_4_^+^ ↔ 4N_2_ + 5H_+_ + 3CH_4_ + 9H_2_O	24
100	HCO^−^_3_ + NH_4_^+^ ↔ NO^−^_3_ + H^+^ + CH_4_	8
101	HCO^−^_3_ + 5H^+^ + 4HS^−^ ↔ 4S^0^ + CH_4_ + 3H_2_O	8
102	HCO^−^_3_ + HS^−^ + H_2_O ↔ SO_4_^−2^ + CH_4_	8
103	3HCO^−^_3_ + 4S^0^ + 7H_2_O ↔ 4SO_4_^−2^ + 5H^+^ + 3CH_4_	24
104	HCO^−^_3_ + H^+^ + H_2_ ↔ CO + 2H_2_O	2
105	HCO^−^_3_ + 2Fe^++^ + 2H_2_O ↔ 2FeOOH_fer_ + 3H^+^ + CO	2
106	HCO^−^_3_ + 2Fe^++^ + 2H_2_O ↔ 2FeOOH_goe_ + 3H^+^ + CO	2
107	HCO^−^_3_ + 3Fe^++^ + 2H_2_O ↔ Fe_3_O_4_ + 5H^+^ + CO	2
108	HCO^−^_3_ + 2Mn^++^ + 2H_2_O ↔ 2MnOOH_feit_ + 3H^+^ + CO	2
109	HCO^−^_3_ + 2Mn^++^ + 2H_2_O ↔ 2MnOOH_man_ + 3H^+^ + CO	2
110	HCO^−^_3_ + Mn^++^ ↔ MnO_2_ + H^+^ + CO	2
111	3HCO^−^_3_ + H^+^ + 2NH_4_^+^ ↔ N_2_ + 3CO + 6H_2_O	6
112	4HCO^−^_3_ + 2H^+^ + NH_4_^+^ ↔ NO^−^_3_ + 4CO + 5H_2_O	8
113	HCO^−^_3_ + 2H^+^ + HS^−^ ↔ S^0^ + CO + 2H_2_O	2
114	4HCO^−^_3_ + 3H^+^ + HS^−^ ↔ 2H_2_O + SO_4_^−2^ + CO	8
115	3HCO^−^_3_ + H^+^ + S^0^ ↔ SO_4_^−2^ + 3CO + 2H_2_O	6
116	3HCO^−^_3_ + 3H^+^ + CH_4_ ↔ CO + 3CO + 5H_2_O	6
**MAGNETITE AS ELECTRON ACCEPTOR**		
117	Fe_3_O_4_ + 6H^+^ + H_2_ ↔ 3Fe^++^ + 4H_2_O	2
118	Fe_3_O_4_ + 2H^+^ + 2Mn^++^ ↔ 2MnOOH_feit_ + 3Fe^++^	2
119	Fe_3_O_4_ + 2H^+^ + 2Mn^++^ ↔ 2MnOOH_man_ + 3Fe^++^	2
120	Fe_3_O_4_ + 4H^+^ + Mn^++^ ↔ MnO_2_ + 3Fe^++^ + 2H_2_O	2
121	3Fe_3_O_4_ + 16H^+^ + 2NH_4_^+^ ↔ N_2_ + 9Fe^++^ + 12H_2_O	6
122	4Fe_3_O_4_ + 22H^+^ + NH^+^_4_ ↔ NO^−^_3_ + 12Fe^++^ + 13H_2_O	8
123	Fe_3_O_4_ + 7H^+^ + HS^−^ ↔ S^0^ + 3Fe^++^ + 4H_2_O	2
124	4Fe_3_O_4_ + 23H^+^ + HS^−^ ↔ SO4^−2^ + 12Fe^++^ + 12H_2_O	8
125	3Fe_3_O_4_ + 16H^+^ + S^0^ ↔ SO_4_^−2^ + 9Fe^++^ + 8H_2_O	6
126	3Fe_3_O_4_ + 18H^+^ + CH_4_ ↔ CO + 9Fe^++^ + 11H_2_O	6
127	4Fe_3_O_4_ + 23H^+^ + CH_4_ ↔ HCO^−^_3_ + 12Fe^++^ + 13H_2_O	8
128	Fe_3_O_4_ + 5H+ + CO ↔ HCO^−^_3_ + 3Fe^++^ + 2H_2_O	2
**PYROLUSITE AS ELECTRON ACCEPTOR**		
129	MnO_2_ + 3H^+^ + H_2_ ↔ Mn^++^ + 2H_2_O	2
130	MnO_2_ + 2Fe^++^ + 2H_2_O ↔ 2FeOOH_fer_ + 2H^+^ + Mn^++^	2
131	MnO_2_ + 2Fe^++^ + 2H_2_O ↔ 2 FeOOH_goe_ + 2H^+^ + Mn^++^	2
132	MnO_2_ + 3Fe^++^ + 2H_2_O ↔ Fe_3_O_4_ + 4H^+^ + Mn^++^	2
133	3MnO_2_ + 4H^+^ + 2NH_4_^+^ ↔ N_2_ + 3Mn^++^ + 6H_2_O	6
134	4MnO_2_ + 6H^+^ + NH_4_^+^ ↔ NO^−^_3_ + 4Mn^++^ + 5H_2_O	8
135	MnO_2_ + 3H^+^ + HS^−^ ↔ S^0^ + Mn^++^ + 2H_2_O	2
136	4MnO_2_ + 7H^+^ + HS^−^ ↔ SO_4_^−2^ + 4Mn^++^ + 4H_2_O	8
137	3MnO_2_ + 4H^+^ + S^0^ ↔ SO_4_^−2^ + 3Mn^++^ + 2H_2_O	6
138	3MnO_2_ + 6H^+^ + CH_4_ ↔ CO + 3Mn^++^ + 5H_2_O	6
139	4MnO_2_ + 7H^+^ + CH_4_ ↔ HCO^−^_3_ + 4Mn^++^ + 5H_2_O	8
140	MnO_2_ + H^+^ + CO ↔ HCO^−^_3_ + Mn^++^	2

* *Aqueous forms were used for O_2_, N_2_, CO, H_2_, etc*.

** *Mineral formulas are as follows: ferrihydrite, FeOOH_fer_; goethite, FeOOH_goe_; magnetite, Fe_3_O_4_; feitknechite, MnOOH_feit_; manganite, MnOOH_man_; pyrolusite, MnO_2_*.

### DNA extraction and 16s rRNA gene sequencing

DNA was obtained from filters and biofilms using a modified phenol-chloroform extraction within 30 days of each sample collection. This included three rounds of physical (freeze, thaw, vortex) and chemical (lysozyme) disruption of the cell wall prior to phenol-chloroform extraction. Controls during DNA extraction and polymerase chain reaction (PCR) confirmed that there were no contaminating nucleic acids during the extraction process (data not shown). Whole genomic DNA was sent to Molecular Research DNA (Shallowater, TX, USA). Primers 515 forward and 806 reverse (Caporaso et al., 2011) were used to amplify the hypervariable 4 region of the 16S rDNA gene according to previously described methods (Dowd et al., 2008). Following amplification, all PCR products from different samples were mixed in equal concentrations and purified using Agencourt Ampure beads (Agencourt Bioscience Corporation, MA, USA). Samples were sequenced on Roche 454 FLX titanium instruments using recommended reagents and following manufacturer's guidelines.

Sequences were analyzed using the programs QIIME (Caporaso, 2010) and Mothur (Schloss et al., 2009), and compared against the SILVA 111 database (Quast et al., 2012). Sequences were quality filtered by removing from the dataset any sequence that did not have an exact match to the proximal primer, contained fewer than 200 or greater than 350 bases, had more than one ambiguous nucleic acid base (Ns), or had a quality score less than 25. Sequences were clustered using the USEARCH algorithm (Edgar, 2010) at a 97% identity cutoff, and taxonomy was assigned to the lowest possible level using the SILVA 111 database (www.arb-silva.de). It is important to note that these data are not strictly quantitative, and thus all percentages reported below should be taken as relative abundance only. All statistical analyses (PCA etc.) were performed using the Matlab® Statistics Toolbox.

## Results

### Sample locations

**Figure 1:**
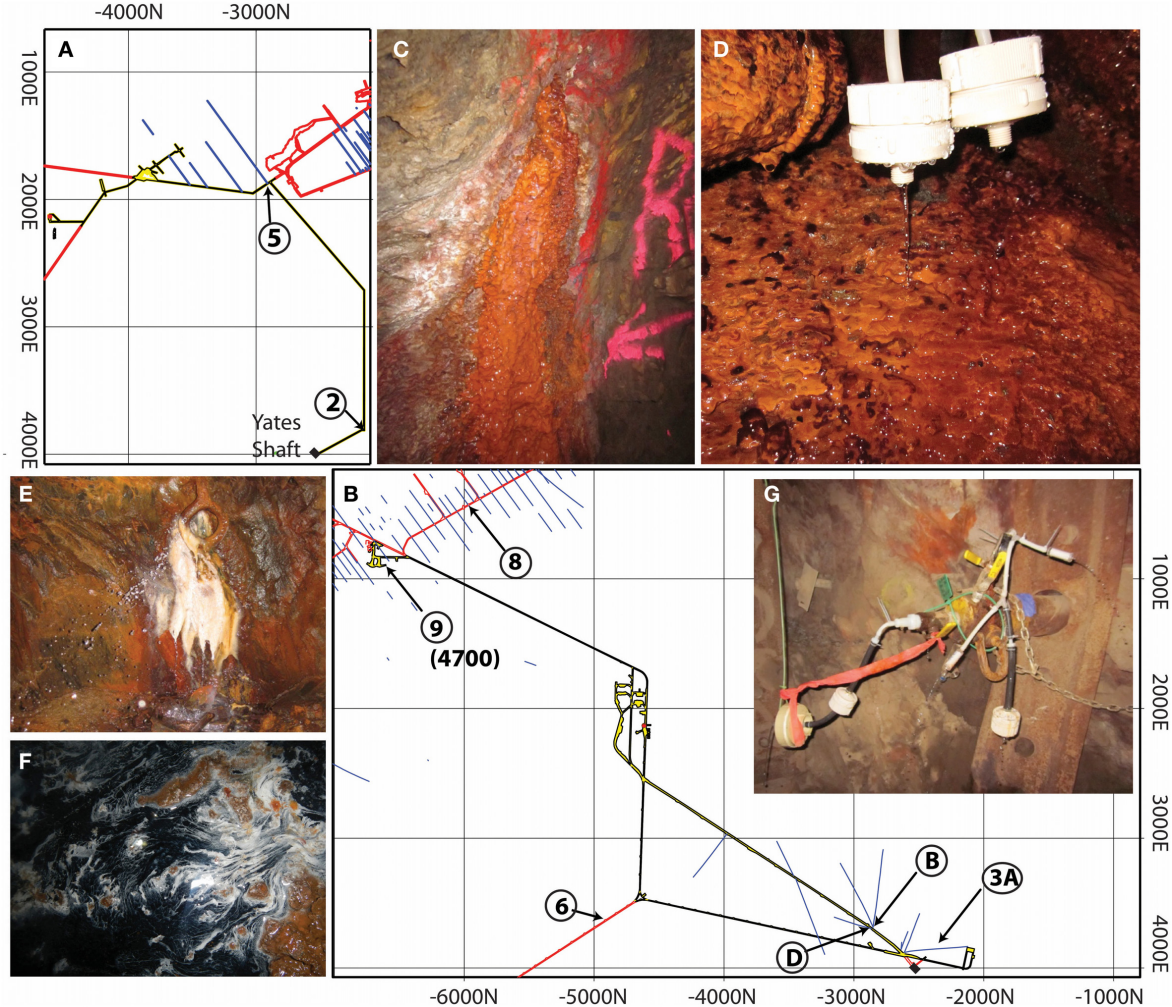
**Plan-view map of sample sites and photographs of sample sites [2 (D), 5 (C), 8 (E), 6 (F), and B (G)]**. Sample locations within SURF. **(A)** Maps of the 800 ft level and **(B)** 4850 ft level showing sampling locations in circles, boreholes in blue, and tunnels in black, red, and yellow. The numerical grid is an internal reference coordinate system in feet. **(C)** Borehole 5 with cascade of iron oxide rich biofilm extending from the source to the tunnel floor below. **(D)** Close up of borehole 2 biofilm showing in-line filtering apparatus and microterraced biofilms. **(E)** Photo of borehole 8 showing water spraying forcefully from the source and thick hanging microbial mats. **(F)** The edge of Pool 6 showing elemental sulfur deposition (white), dark biofilms and mineralization (black), and iron oxides (orange). **(G)** Manifold D during in-line filtration and sampling. Photos were taken by Magdalena R. Osburn.

The eight samples presented in this work were taken from the 800, 4700, and 4850 ft levels from open borehole fluids, capped manifolds, and pools within the mine tunnels at SURF. Replicate analyses from the October 2013 and February 2014 sampling trips are presented for three samples (B, D, and 8). Sampling sites on the 800 ft level, shown in plan view in Figure 1A, feature fluids cascading from diamond drill holes into the mine tunnel, often forming thick accumulations of apparent iron oxides and biofilms extending from the source point (Figure 1C). Microterrace structures of microbial biofilms are present (Figure 1D) as are fine white filaments (not shown). Fluid samples from the deeper levels (see map in Figure 1B) are considerably more diverse. At this depth, boreholes that produce significant flow must be capped with manifolds or plugged with cement to prevent flooding. A subset of these capped sites were opened and sampled (Samples B, D, and 3A, Figure 1G). Site 8 is a diamond borehole that was plugged, but is now producing a large amount of fluid (~2 L/min) through a fracture in the seal (Figure 1E). The last two sampling sites are from pools that are presumably fed with reducing fluids through fissures in the mine walls (Sites 6 and 9, Figure 1F).

### Geochemistry of subsurface fluids

The solute chemistry of the sampled fluids reflects the diversity of environments within SURF and the history of water-rock-microorganism interactions. Field measurements (Table 2) reveal a narrow range of temperature (10–32.8**°**C) and pH (6.55–8.46), but sharp differences in redox chemistry, documented with ORP measurements (79 to -292 mV) and Σ S^2−^ (0.00–2778 μg/L). Significant ranges are also observed in some nutrient concentrations, with nitrate and ammonium varying from below detection limit (bdl) to very high levels (~5 and 0.5 mg/L, respectively). Phosphate concentrations were consistently low but detectable (up to 0.11 mg/L), and nitrite was bdl at all sites. Major anion and cation concentrations also show significant variability (Table 3). Sulfate (96–4400 mg/L) is the dominant anion in all samples, followed by chloride (13–219 mg/L); bicarbonate levels (2.6–15 mM) are elevated at most locations. In most samples, Na^+^ is the dominant cation (27–2125 mg/L), but concentrations of Ca^2+^ (6–456 mg/L) and Mg^2+^ (5–90 mg/L) are as high or higher than Na^+^ at the 800′ level sites. Dissolved gas concentrations are reported in Table 4. Hydrogen is universally low but detectable in some samples (0.01–1.2 nM). In contrast, He was detected in all samples from the 4850′ level except Pool 9, ranging from 0.26 to 8.6 nM. Dissolved CO_2_ concentrations range from 131 to 1212 nM and correlate well with HCO^−^_3_ concentrations. Hydrocarbon gasses co-occur, although methane is more abundant than ethane by a factor 8–202.

**Table 2:** **Field geochemical measurements**.

**Site**	**Level**	**T (°C)**	**pH**	**ORP (mV)**	**cond (uS)**	**TDS (ppm)**	**DO mg/L**	**S^2−^ ug/L**	**Fe^2^+ mg/L**	**NO^−^_3_ mg/L**	**NH_3_ mg/L**	**SiO_2_ mg/L**	**Mn mg/L**	**PO_4_ mg/L**
St2_Oct	800	10	6.55	0	1008	715.7	1.20	7.0	2.50	1.40	0.08	0.00	0.30	bdl
St5_Oct	800	12.4	7.7	127	608.5	422.1	6.10	32.0	0.31	2.20	0.03	9.90	bdl	0.04
St6_Feb	4850	21.2	8.13	292	4757	3679	0.48	2778.0	0.04	4.77	0.37	23.00	bdl	0.11
St8_Oct	4850	30.9	8.06	138	1669	1168	1.40	305.0	bdl	3.10	0.47	22.90	0.60	0.08
St8_Feb	4850	32.8	8.46	264	1586	1105	2.10	382.0	0.01	1.57	0.48	6.00	0.20	0.10
St9_Feb	4700	26.9	8.28	79	1848	1313	5.90	0.0	0.00	0.57	0.01	6.70	0.20	0.07
ManD_Oct	4850	20.4	7.15	193	3206	2393	0.62	28.0	7.06	bdl	0.04	12.90	3.70	0.04
ManD_Feb	4850	17.6	7.73	235	2483	1835	0.09	130.0	2.25	0.64	0.01	11.50	bdl	0.02
ManB_Oct	4850	22.4	7.88	275	7975	6471	2.30	36.0	0.74	bdl	0.10	3.60	0.70	0.03
ManB_Feb	4850	23	7.96	276	7985	6459	0.23	83.0	3.02	1.47	0.09	10.20	bdl	0.00
Man3A_Feb	4850	19.9	7.66	200	7863	6403	2.8^*^	64.0	2.42	bdl	0.06	15.50	0.50	0.08

* *value represents a maximum, sample stored prior to analysis*.

**Table 3:** **Major anions and cations**.

**Site**	**Fl^−^ (ppm)**	**Cl^−^ (ppm)**	**SO^−^_4_(ppm)**	**Sr (ppm)**	**Mg (ppm)**	**Al (ppm)**	**Mn (ppm)**	**Na (ppm)**	**Ca (ppm)**	**Li (ppm)**	**K (ppm)**	**HCO^−^_3_mM**
St2_Oct	0.66	12.54	304.37	3.2	34.0	0.2	0.2	26.6	113.0	0.1	5.2	4.83
St5_Oct	0.76	18.85	96.39	1.4	27.3	0.2	0.1	34.0	45.8	0.0	4.0	6.16
St6_Feb	6.35	85.17	1994.06	6.8	39.8	0.1	0.2	2125.0	56.7	1.3	19.0	8.86
St8_Oct	5.54	22.12	177.45	1.0	5.4	0.1	0.0	329.0	6.1	0.3	12.3	15.01
St8_Feb	2.88	30.95	171.31	1.3	6.2	0.1	0.0	1297.0	8.1	0.3	11.9	12.40
St9_Feb	1.60	25.31	362.22	0.6	59.0	0.1	0.0	301.0	39.9	0.2	12.5	12.62
ManD_Oct	10.12	73.68	1057.29	4.6	16.2	0.2	0.5	527.0	257.7	0.7	24.7	13.50
ManD_Feb	10.79	73.20	590.50	2.7	9.3	0.1	0.1	1442.0	105.9	0.5	13.7	12.45
ManB_Oct	2.97	216.39	4069.20	12.0	90.0	bdl	0.5	1388.0	346.0	2.4	17.0	2.63
ManB_Feb	0.95	219.15	4390.06	20.1	85.4	0.1	0.6	1891.0	450.0	3.9	24.0	2.63
Man3A_Feb	1.14	201.37	4358.07	21.0	78.2	0.1	0.7	1733.0	456.0	3.8	28.9	2.74

**Table 4:** **Dissolved gas composition (nM)**.

**Site**	**He**	**H_2_**	**CO_2_**	**CH_4_**	**ethane**	**CO**	**Propane**
St2_Oct	0.00	0.00	655.23	0.36	0.00	0.29	0.00
St5_Oct	0.00	0.00	517.82	0.00	0.00	0.33	0.00
St6_Feb	0.26	0.01	131.09	15.34	0.53	0.00	0.10
St8_Oct	1.44	0.00	845.42	38.85	0.71	0.15	0.00
St8_Feb	2.87	1.23	975.92	37.98	1.40	2.37	0.22
St9_Feb	0.00	0.01	124.93	0.08	0.01	0.06	0.00
ManD_Oct	1.78	0.00	1212.52	22.09	0.15	0.22	0.00
ManD_Feb	1.62	0.10	187.29	4.97	0.08	0.07	0.03
ManB_Oct	4.12	0.00	177.89	278.50	1.38	0.00	0.00
ManB_Feb	8.61	0.54	64.48	290.23	2.45	0.00	2.27
Man3A_Feb	8.48	0.00	417.79	372.55	4.03	0.00	1.94

### Energetics

**Figure 2:**
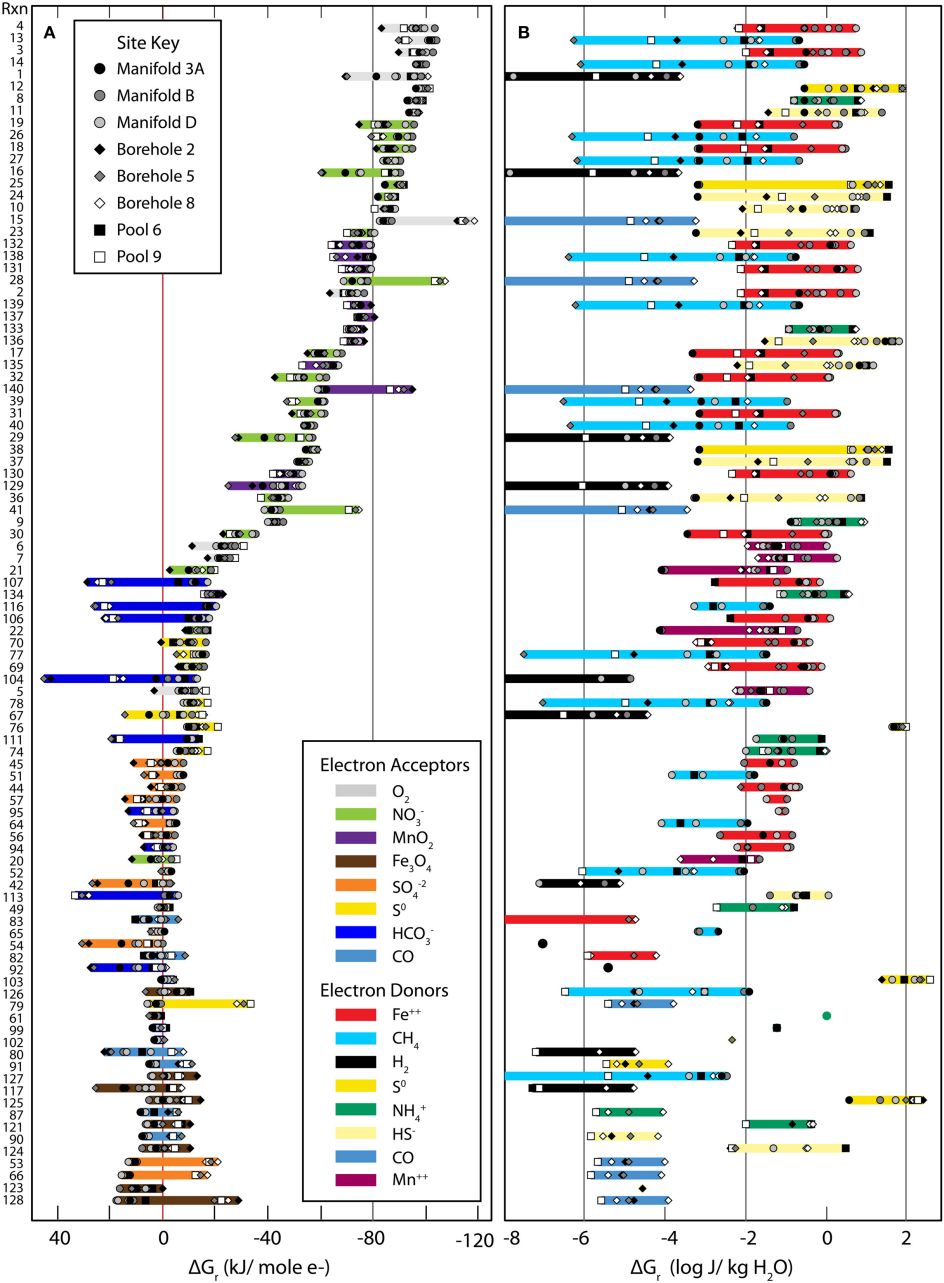
**Gibbs Energy yields from all reactions considered in this study (see Table 1)**. Gibbs energies of the reactions listed in Table 1 referenced by the numbers on the far left side. The small gray-scale symbols refer to values of Δ*G_r_* calculated at individual sites. The left hand panel **(A)** shows Gibbs energies in units of kJ per mole of electron transferred, kJ (mol e^−^)^−1^. The range of Δ*G_r_* values is highlighted by bands whose color corresponds to the electron acceptor in the reactions. The right hand panel **(B)** shows Gibbs energies of reaction as energy densities, Joules per kg of water, J (kg H_2_O)^−1^. The highlighted bars are color coordinated with the electron donor in the reactions.

Values of Gibbs energies for 140 redox reactions (see Table 1) were calculated with Equations (1–3) using geochemical data given in Tables 2–4. Of these reactions, 98 were exergonic in at least one sampling locality, and in Figures 2A,B, the Gibbs energies for each site are represented in kJ per mole of electron transferred and in Joules per kg of water (energy density), respectively. The order from top to bottom in Figure 2 is from most exergonic (~100 kJ/mole e^−^) to least exergonic (near 0 kJ/mole e^−^) determined for the Manifold B site (dark gray circles). Colored horizontal bars indicate the electron acceptor and show O_2_, NO^−^_3_, and MnO_2_ (in gray, green, and purple) are the most exergonic whereas Fe_3_O_4_, SO^2−^_4_, S^0^, and CO (in brown, orange, yellow, and light blue) provide the least amount of energy, ranging from little to none. Sample locations plot near one another, suggesting similar energetic potential between sites. In Figure 2B, Gibbs energies are recast as energy densities using Equation (3). Here the colored horizontal bars indicate the electron donor of each reaction. In this view, energy yield is high using S^0^, HS^−^, NH^+^_4_, Fe^++^, and MnO_2_, but low using CH_4_, CO, H_2_. The distribution between sites is also very large, with individual reactions ranging from highly exergonic to not at all from one site to the other (e.g., Rxn. #27 at site B vs. 3A). The differences between presentations of Gibbs energies and sources of variability are discussed below.

### Overview of microbial diversity

**Figure 3:**
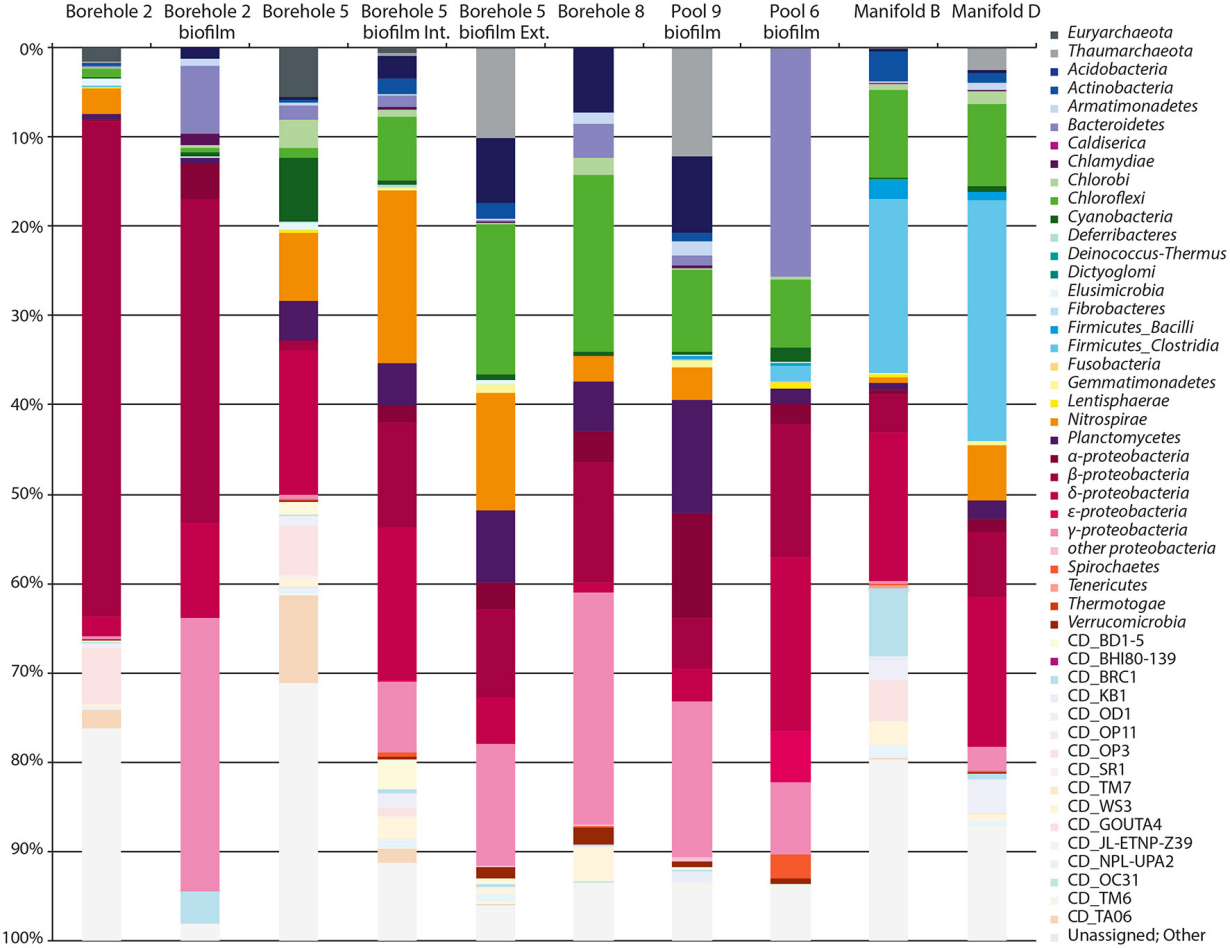
**Phylum level microbial diversity at sample locations**. Phylum-level phylogenetic diversity of each SURF sample site. *Proteobacteria* and *Firmicutes* are further divided into classes. Candidate phyla and OTUs for which no taxonomic assignment could be made are shown in pastel colors.

A total of 27 established bacterial and archaeal phyla along with 16 candidate phyla were detected in the 10 water and biofilm samples (Figure 3). All sites are dominated by Bacteria; Archaea are limited to 5% *Euryarchaeota* in Borehole 5, and 10, 12, and 2.5% *Thaumarchaeota* in Borehole 5, Pool 9, and Manifold D, respectively. No *Crenarchaeota* were observed in this study. While a slight primer bias against Archaea is contributing to the observed abundance patterns, this bias should not more specifically exclude the *Crenarchaeota*. In addition, the general dearth of Archaea was confirmed with sequence independent methods, such as intact polar lipid analysis (data not shown).

Within the Bacteria, the *Proteobacteria* (18–81%) tend to dominate at all sites. However, the class-level breakdown varies from almost exclusively *Betaproteobacteria* (55% in Borehole 2) to including *Deltaproteobacteria* (17–19% in Boreholes 5 and 6, and Manifolds B and D) and/or *Gammaproteobacteria* (26% in Borehole 8). Also common are the *Nitrospirae* (8 of 10 samples, with up to 19% at Borehole 5 internal biofilm), the *Chloroflexi* (all 10 sites, with 20% in Borehole 8), and the *Planctomycetes* (all samples, with 4–12% in Boreholes 5 and 8 and Pool 9). The manifold samples (B and D) contain far more *Firmicutes* (*Clostridia*) and *Actinobacteria* than other samples, and Pool 6 is anomalously high (25%) in *Bacteroidetes*. Phyla for which there are no cultured representatives (candidate phyla) comprise a notable percentage of the sequence diversity at most sites (0.3–19%). Unassigned sequences (despite multiple attempts at identification and implementation of multiple pipelines) comprise 2–29% of our libraries, and vary significantly by site. The variability suggests that these taxa are not merely one group for which our pipeline is insensitive, but rather a diversity of unknown organisms. All sequences are accessible on the Short Read Archive (NCBI) database under BioProject PRJNA262938.

## Discussion

### Sources of subsurface fluids

**Figure 4:**
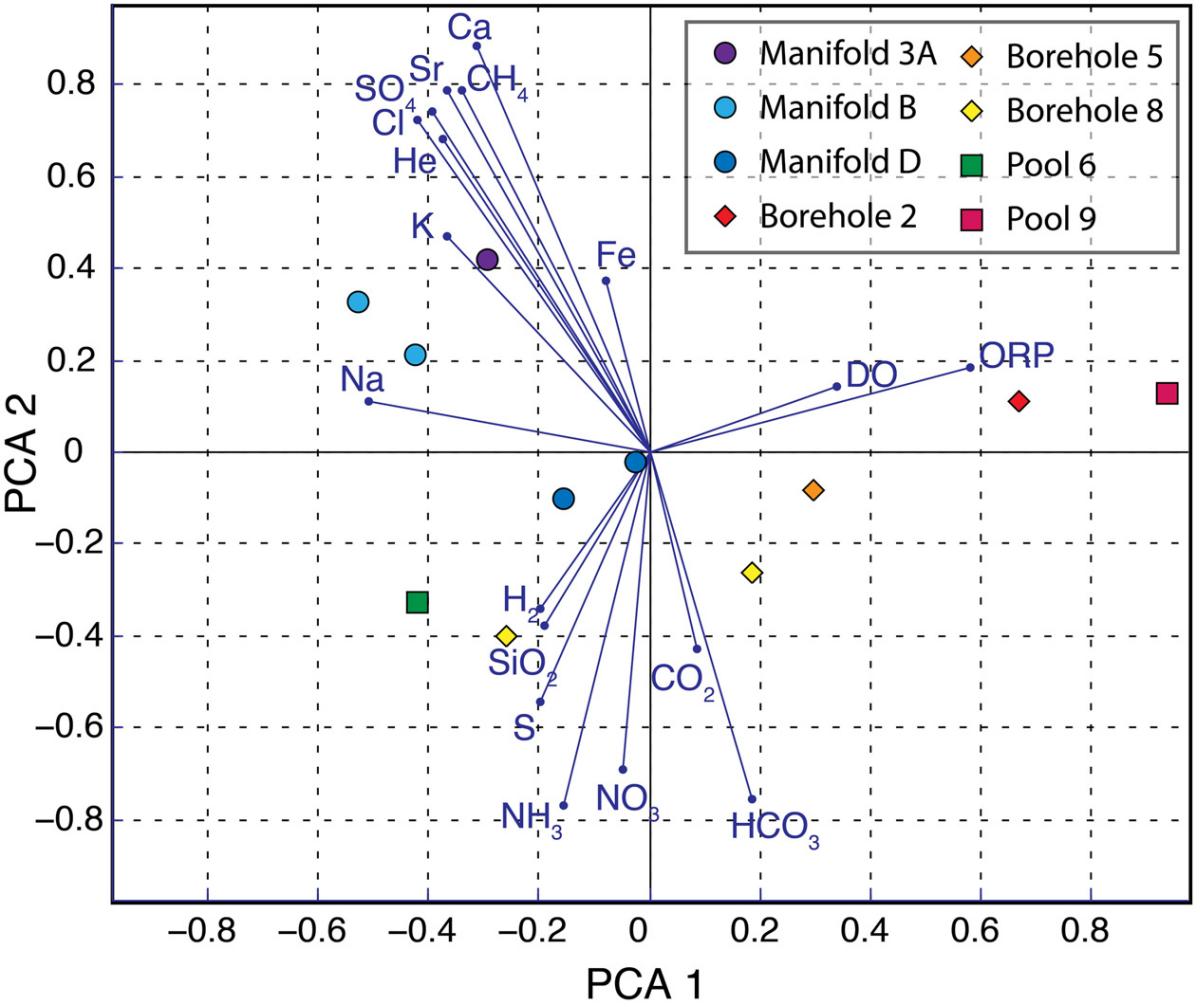
**Principle component analysis of geochemical data**. Principle component analysis of geochemical data from SURF. Sampling sites are shown in colored symbols with manifolds, boreholes, and pools in circles, diamonds, and squares, respectively. Blue vectors illustrate the relationship of each parameter to PCA 1 and PCA 2.

The observed distribution of aqueous species suggests varying levels of water rock interaction, equilibration with the atmosphere, and active cycling of redox sensitive elements between samples. In order to more critically evaluate the relationship between different sites and chemical species, we performed a principle component analysis of all measured geochemical parameters (Figure 4). This analysis reveals strong segregation between the chemistry of fluids taken from manifold sites compared to boreholes. The first principle component (PCA 1) appears to be driven by the dissolved oxygen (DO) concentration and ORP with more oxidizing fluids appearing on the right and reducing fluids on the left. Of the sites that were sampled twice, samples from October plot rightward of those from February, reflecting improved sampling techniques that exclude atmospheric oxygen contamination. PCA 2 groups samples with high total sulfide and ammonium concentration together in the lower half, while samples with high conductivity and total dissolved solids cluster in the upper left hand quadrant (e.g., Manifolds B and 3A). The specific geochemical parameters that also plot in this quadrant (e.g., Sr^2+^, SO^2−^_4_, Ca^2+^, K^+^, Na^+^, Fe^2+^, He) suggest increased water rock interaction. The helium is presumed to derive from alpha decay, indicating prolonged isolation of these fluids from the atmosphere.

### Chemolithotrophy in the homestake mine environment

The energetic calculations carried out in this study suggest that there is a considerable amount of energy to be had from catalyzing chemolithotrophic reactions. However, Gibbs energy can be normalized in multiple ways (e.g., moles of e^−^, moles of reactant, moles of product, per kg water, etc.), each of which highlights different aspects—as has been shown by others (McCollom, 2000; Boettger et al., 2013; LaRowe and Amend, 2014). In Figure 2, Gibbs energy yields are presented in terms of Joules per mole of electrons transferred and also in energy density units of Joules per kg of water. Each of these presentations yields unique structure and identifies different key variables.

Figure 2A presents the standard view of Gibbs energy for each reaction in terms of Joules per mole of electrons as well as the distribution of electron acceptors. We see that the most strongly exergonic reactions utilize O_2_, NO^−^_3_, or MnO_2_ as electron acceptors, similar to the canonical view of electron acceptor potency. Also, there is a relatively tight clustering between different sites, suggesting a broad similarity between energy available at each site, despite large geochemical differences. Outliers are reactions involving dissolved gasses whose activities vary dramatically. From this figure we can see which reactions are most exergonic if sufficient reactants are available. In contrast, Figure 2B presents Gibbs energy in terms of energy density, weighting the theoretical energy yield (shown in 2A) by the availability of limiting reactants. Here, the difference in energy availability for many of the reactions spans several orders of magnitude between sites and some electron donors (S^0^, HS^−^, NH^+^_4_, and Fe^2+^) yield consistently more energy (0.1–100 J/kg water) than others. This presentation of Gibbs energy indicates the importance of local geochemistry to energy supply. On this plot, energy yield does not decrease systematically from the top to the bottom of the figure, suggesting different controls than in Figure 2A.

**Figure 5:**
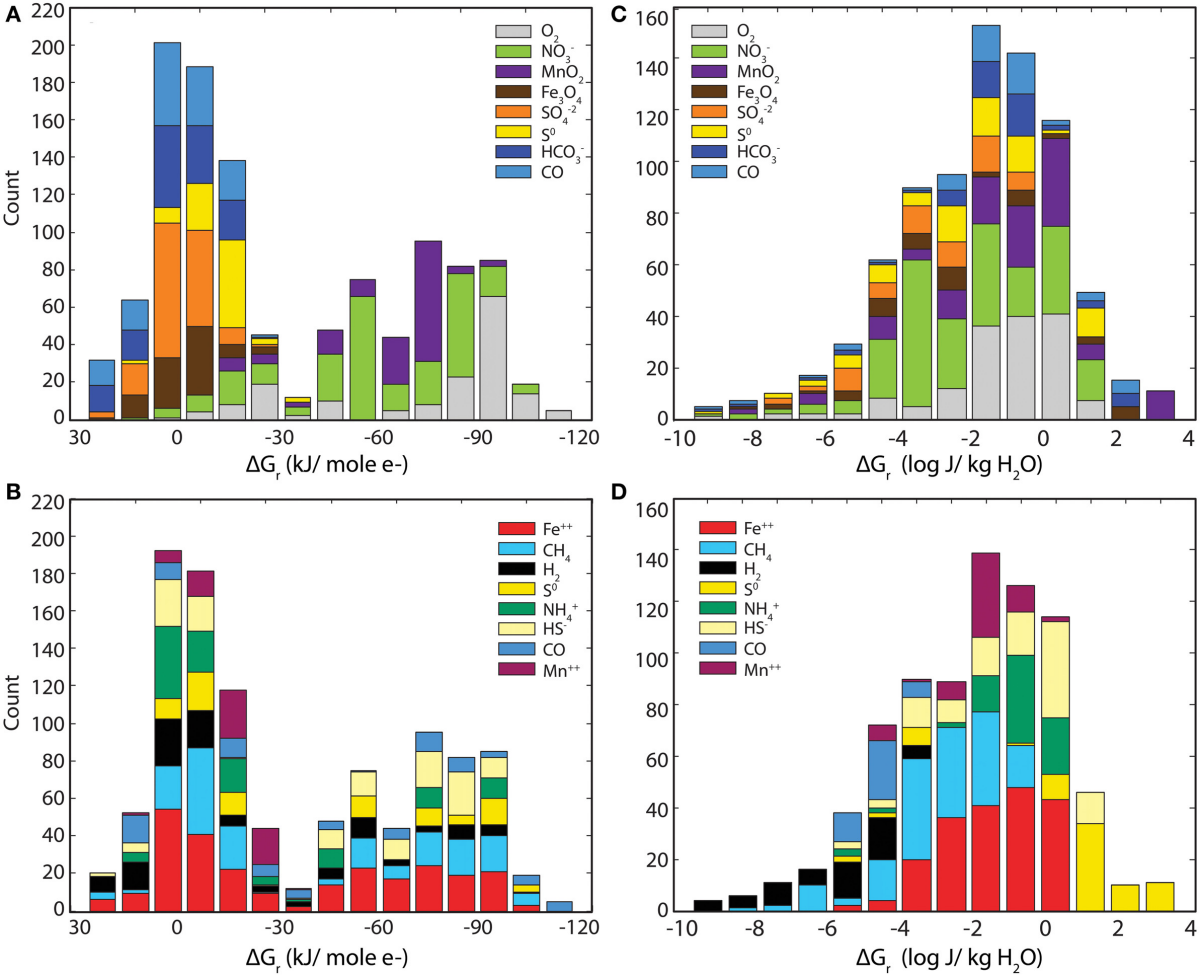
**histograms of Gibbs energies shown in Figure 2**. Histograms of the data from Figure 2 illustrating the relative influence of electron acceptors and donors on Gibbs energy when expressed in multiple ways. The left two panels **(A,B)** show Gibbs energies in units of kilojoules per mole of electron transferred, kJ (mol e^−^)^−1^, where the right panels **(C,D)** show Gibbs energies in units of Joules per kg of water, J (kg H_2_O)^−1^. The upper and lower panels show how various electron donors and acceptors, respectively, are distributed.

To explore this point further, Figure 5 condenses the data points from Figure 2 into histograms that more specifically illustrate the energy available from various electron acceptors (A, C) and electron donors (B, D). Panels A and B (Gibbs energy per mole e^−^ transferred) show a bimodal distribution, with a larger peak centering on 0 kJ (mol e^−^)^−1^ and a smaller, broader peak extended from about −60 to −90 kJ (mol e^−^)^−1^. Note in Panel A (binned by electron acceptor) that reactions with O_2_, NO^−^_3_, and MnO_2_ are more exergonic and are clearly separated from the less exergonic reactions with Fe_3_O_4_, SO^−2^_4_, S^0^, HCO^−^_3_, and CO. In Panel B (binned by electron donor), no trend is observed (Figure 5B). For example, Fe^2+^ oxidation (red bars) spans nearly the entire range from +30 to −100 kJ (mol e^−^)^−1^. Does this mean that aerobes and nitrate reducers should dominate the subsurface at SURF?

Unfortunately for the aerobic microbes, the calculations summarized in panel A and B fail to account for limiting reactants in the systems. Oxygen is a high-energy electron acceptor where available, but where it is limiting this potential cannot be realized. Figures 5C,D recast the Gibbs energy calculations as Gibbs energy densities. Figures 5C,D show a single peak with a maximum at 10^−2^ J (kg H_2_O)^−1^ with tails to very high and low energy yields. Binning this data in terms of electron acceptors shows little relationship to energy (Figure 5C). For example, reactions using nitrate (green) as the electron acceptor range from 10^−10^ to 10 J (kg H_2_O)^−1^. Conversely, structure emerges when the calculated energies are binned by electron donor (Figure 5D), with clearly defined profitable (e.g., S^0^, NH^+^_4_) and poor (H_2_, CO) electron donors. Comparison of the left and right sides of Figure 5 reveals the distribution of molar vs. volumetric Gibbs energy. This analysis illustrates the value of scaling Δ*G_r_* calculations by the limiting reactant for a more realistic view of potential metabolisms. In previous studies, energy density appears to show a stronger relationship to *in situ* microbial communities than unweighted energetic calculations (McCollom, 2000; LaRowe and Amend, 2014). The relationship between energy yield and the corresponding identities of microbial populations is discussed below.

The oxidation of sulfur, sulfide, ferrous iron, ammonium, and the reduction of manganese oxides are strong *in situ* energy sources, but how does energy yield vary by site or with temperature? Oxidation of reduced sulfur species yields large amounts of energy per kg H_2_O at all sites [10^−3^–10^3^ J (kg H_2_O)^−1^], regardless of electron acceptor or temperature (Figure S1). Sulfide oxidation coupled to methanogenesis, the reverse of the consortia mediated anaerobic oxidation of methane (AOM) (Orphan, 2001), is highly exergonic at all sites (#102). A similar consortium of methanogens and sulfide oxidizing bacteria could potentially take advantage of this large energy yield in this system. In addition, iron oxidation is a visibly important process within the SURF environment, resulting in iron-rich biofilms at Boreholes 2 and 5 as well as Pool 9 (see Figure 1 for images), and our calculations confirm significant energy yield for iron oxidation to ferrihydrite and goethite (Figure S2**)**. Mineralogy influences these calculations with higher Gibbs energies for the more crystalline products (Goethite > Ferrihydrite). The relative energy yield between sites is determined by the concentration of ferrous iron rather than the oxidant supply (Manifold D >> Pool 9) and increases with temperature. Ammonium oxidation is another significant source of energy, particularly coupled to O_2_, S^0^, or MnO_2_ reduction (Rxns. # 8, 74, 133; Figure S3). Exergonicity is controlled by the concentration of ammonium with high ammonium sites (e.g., Borehole 8 and Pool 6) yielding energy densities consistently one to two orders of magnitude higher than those with low ammonium concentrations (e.g., Pool 9 and Manifold D). In this case, a negative correlation between energy yield and temperature is seen with higher energy density present at lower temperatures. Finally, manganese oxidation is exergonic at all sites (Rxn. #7, Figure S4), with Gibbs energy yields on par with that of ammonium oxidation and little relationship with temperature.

Clearly, there are many possible exergonic catabolic strategies in the SURF system; however, a number of commonly discussed subsurface metabolisms are absent from this list. Perhaps the most commonly cited subsurface energy source is hydrogen (Nealson et al., 2005), which is an attractive reductant because it has abiogenic sources that are decoupled from surface processes such as the radiolysis of water (Lin et al., 2005; Blair et al., 2007). Hydrogen oxidation with oxygen is a highly exergonic reaction when viewed per mole of electrons (Rxn. # 1; Figure 2 or Figure S4A); however, hydrogen concentrations are simply too low in this system to produce significant energy density (Figure S4B). It is possible that hydrogen oxidizers are consuming hydrogen rapidly as it is generated, creating a cryptic cycle. While it is difficult to evaluate cryptic cycling for this or really any metabolisms using the methodologies described above, molecular data can shed some light on these potential cycles, (discussed below). In addition to hydrogen oxidation, methanogenesis, sulfate reduction, and iron reduction were also considered as potential sources of energy in SURF sites (Rxn. # 92, 42, 117; Figure S4), but were found to yield little to no energy and only at the lowest temperatures. These results contrast with observations of sulfate reducing phyla and probable biogenic methane, but it should be noted that these calculations were performed for only chemolithotrophic reactions.

### Comparison to *in situ* microbial populations

Now that there is compelling evidence for metabolisms that could yield significant amounts of energy in this environment, it is incumbent to ask if microbes capable of mediating these metabolisms are in fact present. Microbes closely related to known chemolithotrophs (based on 16S rRNA gene sequences) are found in abundance; however, the scope of this analysis is incomplete, because many sequences are from uncharacterized strains. To identify likely physiologies, sequencing data were analyzed at the family level for each site, and the families were classified as physiotypes based on the predominant metabolism of cultured representatives as identified in Bergey's Manual of Systematic Bacteriology and in recent publications of newly defined or cultured groups by Iino et al. (2010), Yamada (2006), Bollmann et al. (2014), Chivian et al. (2008). Where metabolisms were unknown (such as for the unclassified groups, Candidate Phyla, or OTUs with only coarse phylogenetic affiliation), the family was placed into the physiotype marked “?”. At some sites this bin constitutes >50% of the OTUs sampled (Borehole 2), whereas in others it is <10% (Pool 6 biofilm). When the family level proved too metabolically diverse to categorize, the family was put into the physiotype labeled “mixed.” The group labeled “P/S?” consists of organisms related closely to oxygenic or anoxygenic photosynthesizers that are unlikely to be mediating these metabolisms in the dark mine environment.

**Figure 6:**
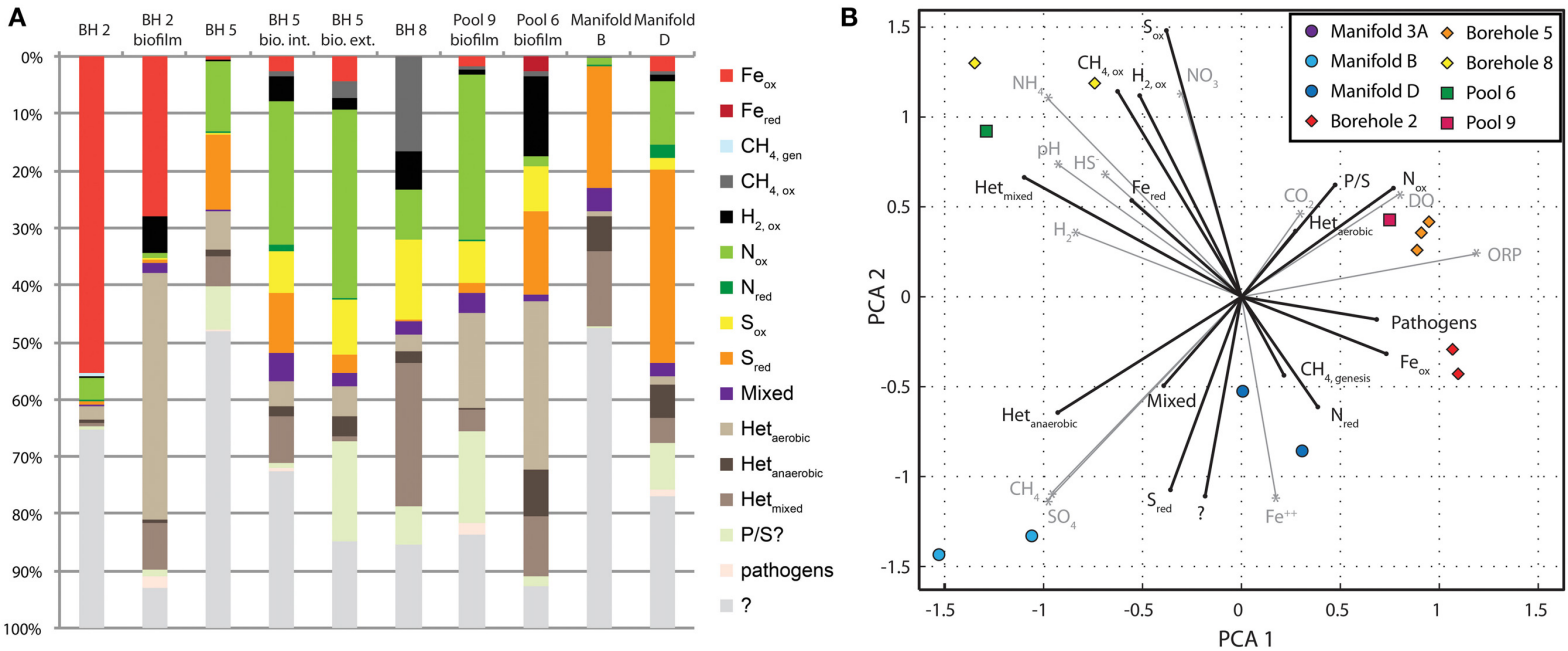
**Physiotypes and their relationship to geochemistry**. Comparison of microbial metabolisms and geochemistry at all SURF sites. **(A)** A bar chart illustrating binned microbial physiotypes at the family level of identified sequences. **(B)** Principle component analysis of physiotype abundance (black vectors) and metabolically important geochemical parameters (gray vectors) by site.

Figure 6A illustrates the distribution of physiotypes within the sampling locations of SURF. At the largest scale it is clear that there is enormous variability in the potential metabolisms present, which is not surprising given the variety and concentrations of electron donors and acceptors at SURF, and therefore the amount of energy available from a diverse set of catabolic strategies. There are distinct differences between the metabolic potential in filtered fluids vs. biofilm samples, as well as between samples from the 800′ and 4850′ levels. In general, the fluids contain a greater proportion of unclassified metabolisms (and phyla, Figure 3) where biofilms contain more heterotrophic constituents. This observation is consistent with trophic structuring and heterotrophic turnover that would be expected in a microbial biofilm or mat. Heterotrophy in samples that are open to the atmosphere is mostly performed by aerobic heterotrophs whereas borehole sites contain more mixed and anaerobic heterotrophic representatives. A similar switch can be observed in the sulfur system where sulfide oxidation yields to sulfate reduction as the prevailing oxidation state progresses from more oxidizing to more reducing sites.

In most cases, particular families dominate the physiotypes at a given site. The most extreme example of this is the Fe_ox_ physiotypes that are almost exclusively composed of the betaproteobacterial family *Gallionellaceae*, which constitutes 54.4% of sequences in borehole 2 fluid. The energy yield of this reaction at site 2 is correspondingly high, up to 0.3–0.4 J per kg H_2_O (Rxn. # 2, 3). Generally, the abundance of the Fe_ox_ physiotype was correlated with increasing energy density from iron oxidizing reactions (*r* = 0.44, see Figure S5). Only a single known iron reducing bacterium was identified, *Geobacteriaceae* which was found exclusively in pool 6. This is one of the few sites where this reaction is energy yielding (Rxn. #119). The CH_4, ox_ physiotype is dominated by the *Methylococcaceae* and most common in Borehole 8. The energy density for aerobic methanotrophy here is correspondingly high (Rxn. 14, 10^−1.5^ J/kg H_2_O). Other dominant families from potentially chemolithoautophic physiotypes include: H_2, ox_—*Hydrogenophilaceae*, N_ox_—*Nitrospiraceae, Thaumarchaeota*, S_ox_—*Thiotrichaceae, Ectothiorhodospiraceae*, S_red_—*Desulfobacteraceaea, Desulfobulbaceae, Peptococcaceae/Desulforudis*.

Comparison of these physiotype abundances to the energy yields shown in Figure 5 illustrates the correspondence between energy density and resident microbial populations. For instance, organisms capable of oxidizing S^0^ and HS^−^ are found in abundance in locations where there are significant amounts of energy available—per kg H_2_O—for these reactions (Figure S5). The same correlation can be made for iron oxidation and nitrogen oxidation. The presence of sulfate reducing and hydrogen oxidizing bacteria do not agree well with energetic predictions. In the case of hydrogen oxidation, the presence of H_2, ox_ communities where measured hydrogen concentrations are too low to yield significant energy, requires that it is produced and consumed by proximal consortia, and either cannot be accurately measured and/or never accumulates in the system. The occurrence of this process is supported in our dataset by the predominance of the H_2,ox_ physiotype in biofilm as opposed to fluid samples.

To further elucidate the relationship between geochemistry and microbial physiotypes, a principle component analysis of both sets of measurements was performed (Figure 6B). Here, vectors for each physiotype are plotted in black and geochemical species in gray, in a principle coordinate system derived from both systems at each site. Species and physiotypes that co-vary in abundance should point in similar directions, whereas physiotypes that are dependent on two chemical species will appear between the two chemical vectors. SURF sample sites are plotted in symbols and their positions reflect proximity to their constituent physiotypes and chemical species.

In this ordination, sites are broadly distributed across the diagram with clustering between 800′ level samples, manifold sites, and sulfidic sites from 4850′ level. Physiotypes are similarly distributed with clusters of CH_4, ox_, S_ox_, and H_2, ox_ in the upper left, N_ox_ and Fe_ox_ on the right, and S_red_ and Het_anaerobic_ in the lower left. An example of a physiotype that depends on multiple chemical species is Fe_ox_, which appears directly in between the DO and Fe^++^ vectors, illustrating its dependence on both variables. The same is true for CH4_ox_ and N_ox_. This also suggests that O_2_ is the terminal electron acceptor for these metabolisms, rather than NO^−^_3_, SO^2−^_4_—or other oxidants. Aerobic heterotrophy is very closely associated with DO, suggesting that oxygen, not organic matter is limiting in these systems. In contrast, there is a close correspondence between the chemical species HS^−^ and H_2_ and their oxidizing phylotypes S_ox_, and H_2,ox_, respectively, suggesting that reductant supply is the determining factor for these metabolisms. Mixed heterotrophy appears antithetical to anaerobic and aerobic heterotrophy, suggesting that it is a combination of the two rather than a poorly characterized phylum.

The diagram shown in Figure 6B can be used to infer metabolisms for the unknown physiotypes. For instance, in the case of sulfate reduction, it can be seen that this physiotype (S_red_) plots in the lower left, very near to Het_anaerobic_ and “?” physiotypes. Sulfate reduction with hydrogen was shown to be a poor source of energy in this system (see Chemolithotrophy), which seemingly contradicts the abundance of SRBs in a number of sites. Notably, the S_red_ vector does not fall in between H_2_ and SO_4_, and instead appears near that for anaerobic heterotrophy. Perhaps these sulfate reducers are using organic matter rather than H_2_ as an electron donor. Many of the SRBs identified in SURF are capable (and often require) heterotrophic modes of growth, lending support to this idea. The big unknown in these, and in most ecosystems, is the metabolic potential of the unclassified organisms. While this analysis is not detailed enough to suggest a specific metabolism, the location of the “?” physiotype near S_red_ and in the neighborhood of CH_4 genesis_ and Het_anaerobic_ appears to suggest at the very least that these organisms are likely anaerobes. Further efforts to isolate these unknown organisms and characterize their metabolic potential through genomic studies are required to further resolve these questions.

### Energetics in different geochemical environments

This is not the first study to quantify environmental energy availability; previous studies have targeted diverse environments including terrestrial hot springs, submarine hydrothermal systems, igneous marine basement, and marine sediments (McCollom, 2000; Amend et al., 2003, 2011; Shock et al., 2005, 2010; Spear et al., 2005; LaRowe et al., 2008; Vick et al., 2010; LaRowe and Amend, 2014; Teske et al., 2014). While the sites are unrelated, there appears to be an upper limit on Δ*G_r_* of approximately −120 kJ (mole e^−^)^−1^. This value reoccurs in a diversity of environments, including terrestrial hydrothermal systems, marine sediments, marine basement, and shallow marine hydrothermal systems. In different environments, different reactions are the most exergonic: for example, aerobic iron oxidation to magnetite in Yellowstone hot springs evaluated in Shock et al. (2010), the knallgas reaction in Juan de Fuca Ridge basement fluids described in Boettger et al. (2013), and nitrite reduction with H_2_ in an acidic thermal fluid on Vulcano Island (Italy) noted in Amend et al. (2003); several other reactions, however, yield similarly high energy. In the SURF environments described here, the most exergonic reaction is aerobic carbon monoxide oxidation, with a Δ*G_r_* of −118 kJ/mole e^−^. These most exergonic reactions use O_2_, NO^−^_3_, or NO^−^_2_ as the terminal electron acceptor, which is consistent with the canonical electron tower model of microbial metabolism. If this were the whole story, it would have significant and grave implications for subsurface life that is truly isolated from surface processes, not to mention life in the Archean oceans.

In this light, the reversal of fortunes that occurs when Δ*G_r_* is instead presented as energy density is most revealing. Energy yields switch from being very dependent on the electron acceptors O_2_, NO^−^_3_, and MnO_2_ to instead depending on the electron donors S^0^, HS^−^, NH^+^_4_, Fe^++^, and Mn^++^. Notably, this switch is present almost wherever both formulations of Δ*G_r_* are presented. For instance, Boettger et al. (2013) saw energy yield switch from being primarily dependent on O_2_ and NO^−^_3_ to depending on CH_4_ and H_2_ with a range of electron acceptors. Similar trends are observed in LaRowe and Amend (2014) with the energy density emphasis on sulfur species. Similar to this study, they found that energy density calculations correspond more closely to resident populations than kJ (mole e^−^)^−1^ calculations. Viewing energetic potential in terms of energy density may both more accurately represent environmental systems and introduce a number of potentially profitable, surface independent, metabolisms to fuel the DSB.

## Concluding remarks

The SURF laboratory is a portal into the DSB that reveals an array of geochemically, taxonomically, and metabolically diverse microbial communities. This variability and the interrelatedness of geochemical and biological processes have been documented here. This study confirms and contributes to the growing body of knowledge that energetics should be viewed in reference to the limiting reactants in environmental systems. These data often correspond well to the metabolic capability of microbial populations and may be generally more representative of the *in situ* biogeochemical processes. This is substantiated in the current study by presenting both energetic calculations and molecular characterization of microbial populations together. By assigning presumed physiology to taxonomic groups, the predictive power of the energetic calculations can be refined, and in some cases, be used to see what energetic calculations alone cannot. Statistical analysis of these data can be harnessed to generate hypotheses for the function of unknown microbial taxa and cryptic biogeochemical cycles. Replication of this approach at other terrestrial deep subsurface sites will contribute to our understanding of the metabolic activity of these isolated ecosystems and their importance to global biogeochemical cycling.

### Conflict of interest statement

The authors declare that the research was conducted in the absence of any commercial or financial relationships that could be construed as a potential conflict of interest.
