# Supplementary material for: Chemolithotrophy in the continental deep subsurface: Sanford Underground Research Facility (SURF), USA
Source: Front Microbiol. 2014 Nov 12;5:610. doi: 10.3389/fmicb.2014.00610 (PMC4228859; doi:10.3389/fmicb.2014.00610)
Supplement: Supplementary file 1 [file Table1.PDF]

Supplementary Figures:

Figure S1: S oxidation

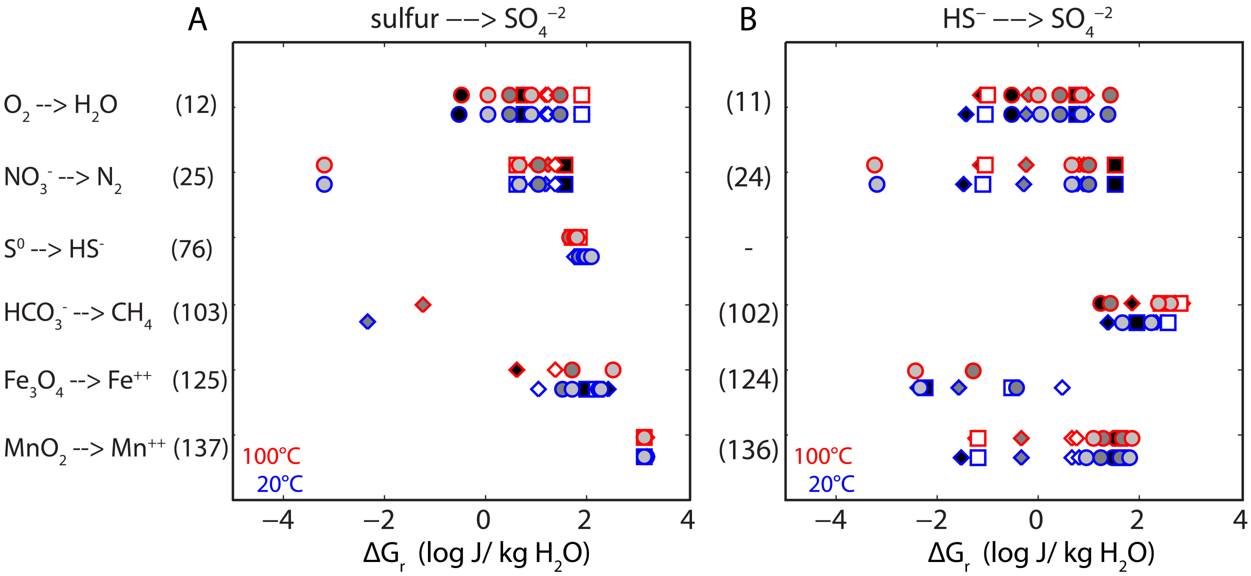

Figure S1: Gibbs energy density, J (kg H_2O)$^{-1}$, of sulfur (A) and sulfide (B) oxidizing reactions. Electron donors and products are shown on the left side and corresponding reaction numbers (**Table 1**) are shown to the left of each panel. Site-specific symbols are as in **Fig. 2** and red and blue rims indicate the results for calculations at 100 and 20°C, respectively.

Figure S2: Iron oxidation

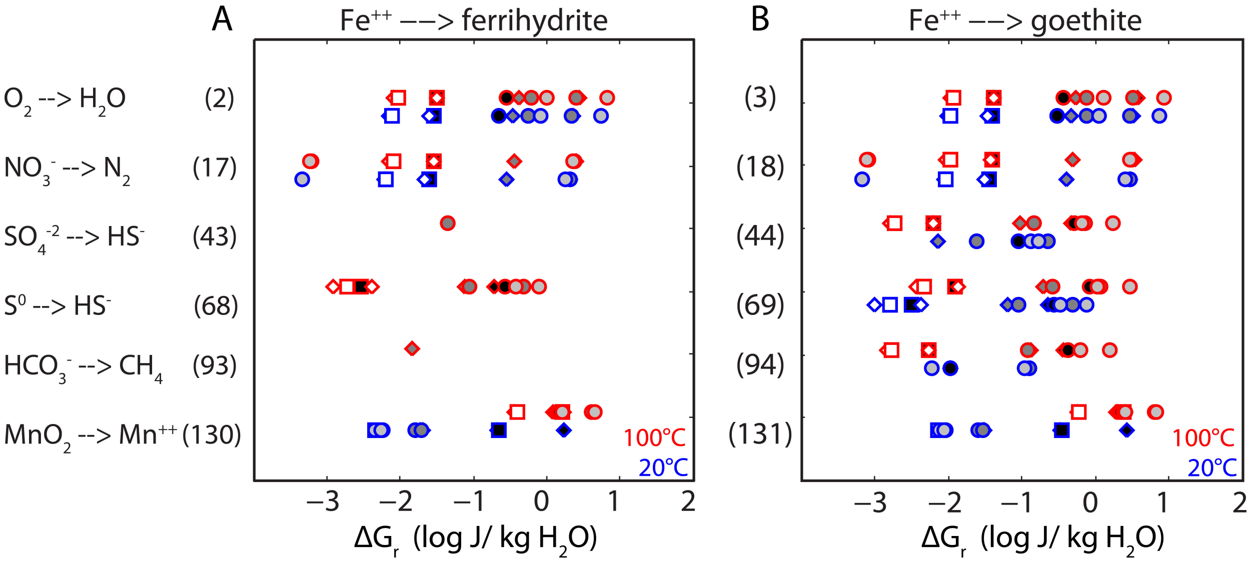

Figure S2: Gibbs energy density, J (kg H_2O)$^{-1}$, of iron oxidation to ferrihydrite (**A**) and goethite (**B**). Electron donors and products are shown on the left side and corresponding reaction numbers (**Table 1**) are shown to the left of each panel. Site-specific symbols are as in **Fig. 2** and red and blue rims indicate the results for calculations at 100 and 20°C, respectively.

Figure S3: Ammonium oxidation

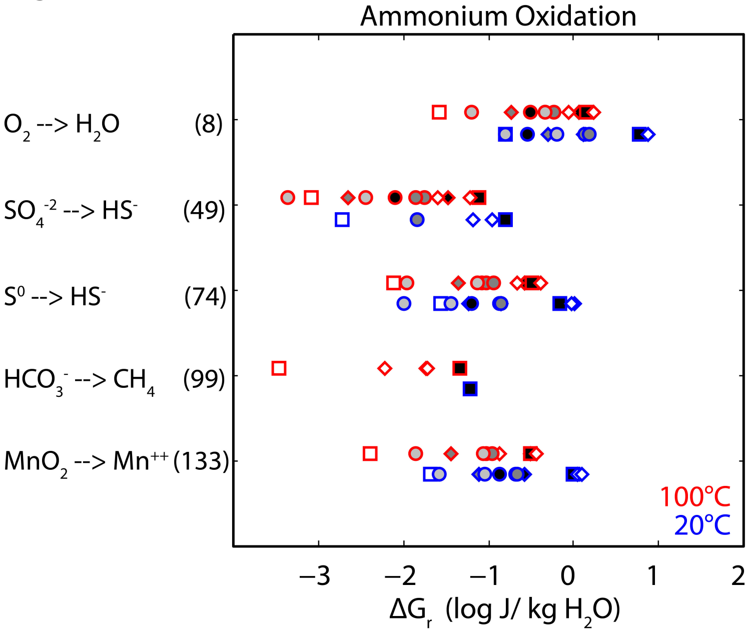

Figure S3: Gibbs energy density, J (kg H_2O)$^{-1}$, of ammonium oxidation for a variety of electron donors. Donor pairs and corresponding reaction numbers are indicated on the left margin. Symbols are as in **Fig. 2** and red and blue rims indicate the results for calculations at 100 and 20°C, respectively.

Figure S4: Common subsurface catabolic reactions

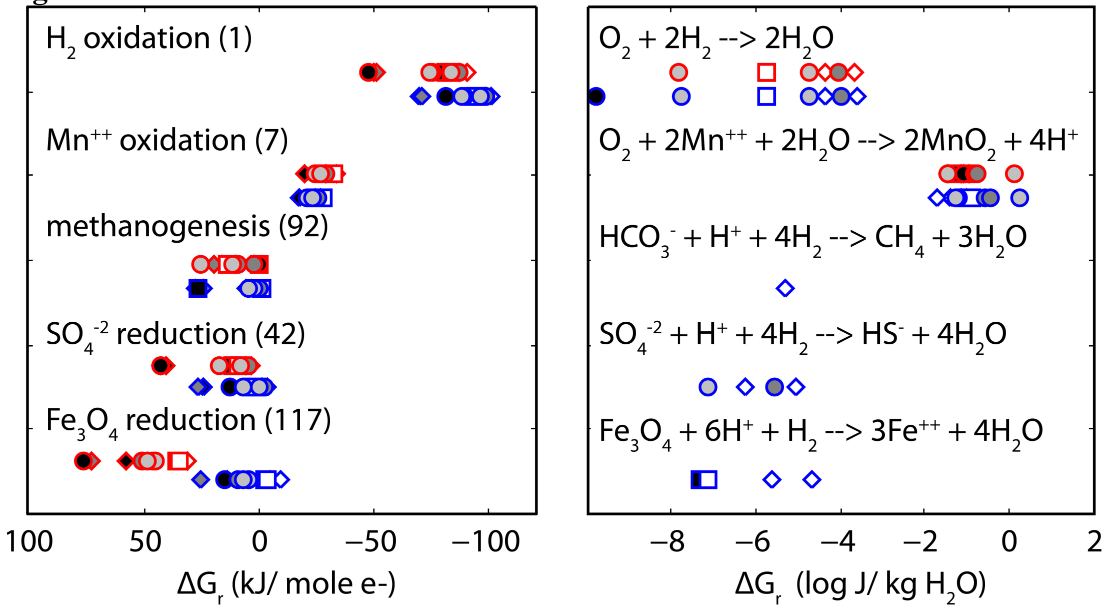

Figure S4: Gibbs energy, shown both per mole of electrons, kJ (mol e^-)$^{-1}$, (left) and energy density, J (kg H_2O)$^{-1}$, (right), for common subsurface chemoautotrophic metabolic reactions. Common names for these catabolisms and reaction numbers are shown on the left panel and full reactions are shown on the right side. Site-specific symbols are as in **Fig. 2** and red and blue rims indicate the results for calculations at 100 and 20°C, respectively.

Figure S5: Correlations between physiotypes and energy density of reaction

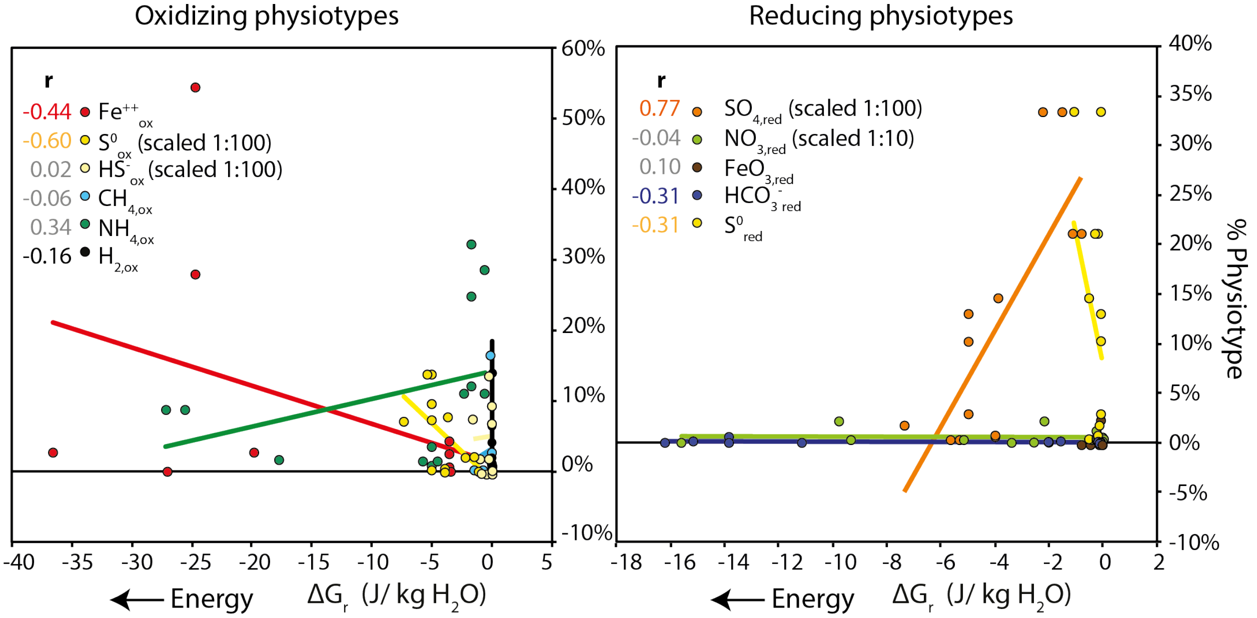

Figure S5: The relationship between predicted energy density and observed physiotypes is tested by correlating energy density for a suite of reactions (e.g. iron oxidation) vs. the abundance of the physiotype (e.g. Fe_{ox}) thought to mediate the reaction. Correlation coefficients (r) are listed in the legend of each figure.
